# Awareness of the Signs, Symptoms, and Risk Factors of Cancer and the Barriers to Seeking Help in the UK: Comparison of Survey Data Collected Online and Face-to-Face

**DOI:** 10.2196/14539

**Published:** 2020-01-17

**Authors:** Katie Connor, Briony Hudson, Emily Power

**Affiliations:** 1 Cancer Research UK London United Kingdom

**Keywords:** neoplasms, surveys and questionnaires, cross-sectional study, awareness, help-seeking behavior, cancer

## Abstract

**Background:**

Cancer is the second leading cause of death globally, causing an estimated 9.6 million deaths in 2018. Low cancer symptom awareness has been associated with poor cancer survival for all cancers combined. The Cancer Awareness Measure (CAM) is a validated, face-to-face survey used since 2008 to measure the UK public’s awareness of the symptoms and risk factors of cancer as well as the barriers to seeking help.

**Objective:**

The aim of this study is to explore whether online data collection can produce a representative sample of the UK population, compare awareness of cancer signs and risk factors and the barriers to seeking help between data collected online and face-to-face, and examine the relationships between awareness and demographic variables.

**Methods:**

Differences in awareness of cancer signs, symptoms, and risk factors among samples were explored while adjusting for demographic differences (age, gender, ethnicity, educational level, marital status, and country of residence) to distinguish the effect of data collection method. Multivariate logistic regression models were used to calculate adjusted odds ratios for recall and recognition of signs and symptoms, risk factors, and barriers to seeking help.

**Results:**

A total of 4075 participants completed the CAM, 20% (n=819) via face-to-face interviews and 80% online (n=3256; agency A: n=1190; agency B: n=2066). Comparisons of data collected using face-to-face interviews and online surveys revealed minor differences between samples. Both methods provided representative samples of the UK population with slight differences in awareness of signs, symptoms, and risk factors and frequency of help-seeking barriers reported.

**Conclusions:**

These findings support a move to online data collection for the CAM. The flexibility afforded will enable the CAM to explore a wider range of issues related to the prevention, early diagnosis, and treatment of cancer.

## Introduction

### Background

Cancer is the second leading cause of death globally, causing an estimated 9.6 million deaths in 2018 [1]. Half of the people diagnosed with cancer in England and Wales survive for 10 years or more, but approximately 4 in 10 cases of cancer in the UK could be prevented [2]. Cancer survival has consistently been reported to be lower in the UK than similar European countries [3,4].

Late-stage diagnosis contributes to excess deaths for bowel [5], breast [6], and lung cancer [7] in the UK. Late diagnosis could be related to low awareness of symptoms, leading to delays in seeking medical help. Low cancer symptom awareness has been associated with delays in seeking medical help and poor cancer survival for all cancers combined [8].

Earlier detection can improve patient experience [9], costs to the National Health Service (NHS) [10], and cancer survival, but it relies partly on prompt presentation [11]. Understanding and potentially improving awareness of cancer signs is an important step in reducing the incidence of late-stage cancer and reducing cancer deaths in the UK.

In 2008, Cancer Research UK, in partnership with University College London, King’s College London, and University of Oxford, developed the Cancer Awareness Measure (CAM) [12]. The CAM is a validated survey designed to measure awareness of signs, symptoms, and risk factors for cancer and potential barriers to seeing a doctor.

Cancer Research UK has used the CAM to collect data biannually from 2008 to 2014 from a representative sample of the UK population via the Office for National Statistics (ONS) Opinions and Lifestyle Survey. Questions in the survey are a combination of recall and recognition questions, designed to assess public awareness. Recall questions are open-ended questions, asking participants to list as many cancer warning signs and risk factors that they can think of. These are followed by recognition questions, where participants are given a list of warning signs and risk factors and asked yes/no do they think these are risk factors or warning signs of cancer.

Data from the CAM indicate that the average number of cancer warning signs recognized by representative samples of the UK population has increased from 6.4 (SD 1.9) in 2008 to 6.8 (SD 1.5) in 2014 out of a possible nine warning signs posed in the survey [13]. Recall of risk factors appears to have followed the opposite pattern, with recall decreasing from a mean 2.2 in 2008 to 2.0 in 2014 [13]. Awareness of cancer signs and risk factors has consistently been found to be lower among men [14,15], younger adults [14], and those from lower socioeconomic groups [14,16,17] or ethnic minorities [15,18].

Although CAM data have traditionally been collected via face-to-face interviews conducted by the ONS, the response rates have declined over the years (from 61% in 2008 to 47% in 2017). This study explores the viability of moving data collection online, a move seen in many large market research organizations. In Great Britain, 90% of households have access to the internet, and 73% of people have accessed the internet with a mobile phone [19]. The benefits of online data collection include lower costs [17], higher data quality [20], and a faster rate of return and lower data entry times [21]. Conversely, the limitations may include sampling issues [21] and differences in sampling methodologies [22].

Although the relationships among questionnaire modality, response rates, and accuracy have been described as complex [23], previous research exploring the impact of data collection method is encouraging. Socially desirable behaviors have been reported to be less likely to be disclosed in interviews than online questionnaires [24], and disease prevalence rates are much closer to known rates when using internet studies compared with data collected over the telephone or face-to-face [25].

### Research Objectives

The primary aim of this study is to identify the extent to which public awareness of cancer and attitudes toward seeking help vary by data collection method (face-to-face vs online data) in adults (aged ≥18 years) in Great Britain. The research objectives are to (1) explore whether online data collection can produce a representative sample of the UK population (differences between samples); (2) compare the awareness of signs, symptoms, and risk factors for cancer, as well as the barriers to seeking help between data collected online and face-to-face (differences in levels of awareness); and (3) explore whether any relationships observed between awareness and demographic variables are consistent across samples (interactions between survey provider and demographic variables).

## Methods

### Participants and Recruitment

#### Face-to-Face Sample

Between January and March 2017, face-to-face data were collected by the ONS via the Opinions and Lifestyle survey. The ONS use stratified probability sampling to select sampling points from a database of 27 million private households in the UK. A random sample of addresses from each sampling point were selected, and interviewers invited one adult respondent from each household to complete the CAM using a face-to-face, computer-assisted interview.

#### Online Samples

Online samples were recruited by two market research agencies. Agency A recruited participants to their online panel via a face-to-face survey. Agency A used a probability-based approach for recruitment, which avoids in-built bias commonly found in online panel sampling methods. Agency B used “active sampling,” in which a subsample of participants were selected from their more than 800,000-member panel based on their age, gender, social class, and education. Agency B panel members are recruited from standard advertising and strategic partnerships with a range of websites.

### Great Britain Population Data

The Great Britain population statistics were taken from the ONS (midyear population estimates, Households and Individuals Internet Access survey), census data, and NHS Digital (Health Survey for England).

### Outcome Measures

Variables collected in the CAM are outlined in Textbox 1. Details of the development and content of the CAM can be found elsewhere [12].

To reduce bias, open-ended questions about signs, symptoms, and risk factors were asked before closed questions. The number of warning signs endorsed or risk factors recognized were summed to produce total scores. Coding manuals were provided to all market research agencies regarding how to code recalled items to ensure consistency.

**Outcome measures.**

**Sociodemographic characteristics**

- We amended the standard ONS demographic questions and adapted these for online samples where necessary: age, gender, educational attainment, ethnicity, country of residence marital status, internet use, and self-reported health status.

**Awareness of signs and symptoms of cancer (recall and recognition)**

- Recall: “There are many warning signs and symptoms of cancer, please name as many as you can think of.”
- Recognition: “Could any of the following be signs of cancer?”: lump or swelling, persistent unexplained pain, unexplained bleeding, persistent cough or hoarseness, persistent change in bowel or bladder habits, difficulty swallowing, change in the appearance of a mole, a sore that does not heal, and unexplained weight loss.

**Awareness of cancer risk factors (recall and recognition)**

- Recall: “What things do you think affect a person’s chance of developing cancer?”
- Recognition: “Could any of the following increase a person’s chance of developing cancer?”: smoking, getting sunburned, exposure to another person’s smoking, drinking alcohol, having a close relative with cancer, being overweight, being older, not eating many fruits and vegetables, not eating enough fiber, eating too much red or processed meat, not doing much physical activity, and infection with HPV (human papillomavirus).

**Barriers to seeing a general practitioner**

- “Which of the following might put you off going to the doctor?”
- Participants were asked to indicate whether any of a range of barriers might put them off seeing a doctor on a 5-point agreement scale from strongly agree to strongly disagree.

### Statistical Analysis

#### Weighting and Sample Differences

Each market research agency provided their own weighting variable to ensure the sample was representative of the Great Britain population and to adjust for nonresponse where possible. Our analyses were carried out using the weighted variable provided by each agency. We did not create a bespoke weighting variable because of the lack of nonresponse data available. See Multimedia Appendix 1 for how each survey provider weighted their data.

Weighted sample demographics were compared between the surveys to explore any differences between the collected samples. Differences between survey responses and Great Britain population statistics were not tested for significance because confidence intervals for Great Britain data were not available.

#### Differences in Levels of Awareness

Differences in awareness of cancer signs and symptoms and risk factors between samples were explored while adjusting for demographic differences (age, gender, ethnicity, educational level, marital status, and country of residence) with the aim of determining the effect of data collection method.

Multivariate logistic regression models were used to calculate adjusted odds ratios for recall and recognition of signs and symptoms, risk factors, barriers to seeking help, and awareness of bowel screening. The outcome variable was binary to show if the responder did or did not recall or recognize signs and symptoms, risk factors, barriers to seeking help, and awareness of bowel screening. Only statistically significant variables were included in the final logistic regression models.

#### Interactions Between Outcomes and Demographic Variables

Interaction terms between survey provider and key demographics (gender, age, education level, marital status, ethnicity, country, long-term health, and internet usage) were added to the awareness models. Whether data collected by different methods varied by demographic variables, while controlling for any differences in sample characteristics between the surveys, was explored.

## Results

### Participants

In total, 4075 participants completed the CAM. Online participants made up 80% (n=3256) of the sample (agency A: n=1190; agency B: n=2066). The remaining 20% (n=819) of participants completed face-to-face interviews.

### Differences Between Samples

The three weighted samples were generally representative of the Great Britain population (Table 1).

**Table 1:** Demographic characteristics by survey provider and compared with Great British population statistics (N=4075).

Demographic		Face-to-face, %	Online, %^a^		Great Britain population, %
		Office for National Statistics (n=819)	Agency A (n=1190)	Agency B (n=2066)	
**Age groups**					
	18-24	10.2	8.4	12.0	15.1^b^
	25-44	33.8	33.4	32.1	32.1
	45-54	18.0	17.9	20.9	17.2
	55-64	15.1	16.9	16.9	13.9
	≥65	22.8	23.1	18.1	21.7
	Missing	—	0.2	—	—
**Gender**					
	Male	49.1	49.9	48.0	49.3
	Female	50.9	50.1	52.0	50.7
**Ethnicity**					
	White	87.9	87.5	92.7	86.0
	Nonwhite	12.1	12.5	7.3	14.0
**Country of residence**					
	England	86.6	84.7	86.3	86.5
	Scotland	8.3	10.2	8.7	8.6
	Wales	5.1	5.1	5.0	4.9
**Higher education qualification**					
	Degree	30.5	26.4	32.2	27.1
	Below degree	42.7	55.7	54.1	44.7
	No qualifications	12.7	15.5	6.6	23.0
	Other	14.1	2.3	5.5	5.2
	Don’t know	—	—	1.6	—
**Marital status**					
	Partner	50.5	62.6	61.7	50.9
	No partner	49.5	37.4	38.3	49.1
**Long-term illness**					
	Very good	37.0	20.1	15.6	Very good/good: 76
	Good	42.0	48.5	47.4	—
	Fair	15.9	23.7	28.3	—
	Bad	3.6	6.4	7.1	Very bad/bad: 7
	Very bad	1.3	1.3	1.6	—
	Refused	0.3	0.1	—	—
**Internet usage**					
	Several times a day	64.2	65.6	79.9	At least once a day: 80
	Once a day	14.3	13.2	13.7	—
	4-6 days a week	3.1	2.9	3.0	—
	2-3 days a week	3.7	4.2	1.6	—
	Once a week	2.1	2.4	0.6	At least weekly: 8
	Less than once a week	1.3	2.2	0.4	Less than weekly: 2
	Never	9.2	9.5	0.8	Did not use in the last 3 months: 10
	Don’t know	0.9	—	—	—
	Refused	1.3	—	—	—

^a^Percentages are weighted using the weighting variable provided by each survey agency; see Multimedia Appendix 1 for more information.

^b^Ages 15-24 years.

The gender split of all three samples largely matched the Great Britain population; however, both online samples were older than the ONS sample and the Great Britain population. Scottish participants were slightly overrepresented by agency A (10.2% vs 8.6% of Great Britain population).

All samples included a higher proportion of white participants than the Great Britain population (Great Britain population: 86%; agency A: 87.5%; agency B, 93%) and reported higher educational attainment. Both online samples had a larger proportion of participants with a partner (agency A: 63%; agency B: 62%) compared with the Great Britain population (50.9%) and were more likely to report being in good health (agency A: 48.5%; agency B: 47.4%; ONS: 42%). Face-to-face participants were less likely to report their health as bad (3.6%; agency A: 6.4%; agency B: 7.1%; Great Britain population: 7%). More than 90% of agency B participants reported using the internet more than once a day compared with 78.5% of face-to-face and 78.8% of agency A participants.

### Differences in Levels of Awareness (Outcomes)

The number of cancer warning signs and risk factors recognized and recalled within each sample are included in Multimedia Appendix 2.

### Cancer Warning Signs

#### Recall of Warning Signs

**Figure 1:**
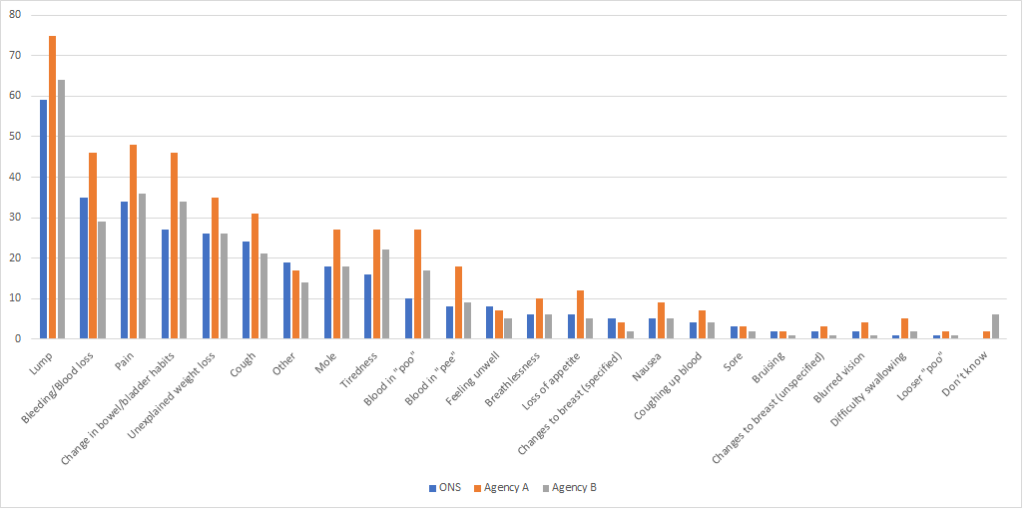
Percentage of participants recalling cancer warning signs.

Agency A participants recalled significantly more signs of cancer than other participants, with a mean recall of five signs of cancer compared with three for both face-to-face and agency B participants. Figure 1 shows the percentage of participants recalling cancer warning signs.

A lump was the most frequently recalled sign in all three samples (agency A: 75.1%, agency B: 64.2%, face-to-face: 58.6%; Figure 1). Compared with face-to-face participants, agency B participants were less likely to recall bleeding or blood loss (29% vs 35%, *P<*.001, OR 0.7, 95% CI 0.6-0.8) and sores (1.5% vs 2.7%, *P*=.003, OR 0.4, 95% CI 0.2-0.8). Agency A participants were more likely than face-to-face participants to recall a lump (75% vs 59%, *P*<.001, OR 2.3, 95% CI 1.8-2.8), pain (48% vs 34%, *P*<.001, OR 1.9, 95% CI 1.6-2.3), bleeding or blood loss (46% vs 35%, *P*<.001, OR 1.5, 95% CI 1.3-1.8), and blood in urine (18% vs 8%, *P*<.001, OR 2.5, 95% CI 1.9-3.3). Participants from both online samples were more likely than face-to-face participants to recall change in bowel or bladder habits (agency A: 46%, *P*<.001, OR 2.9, 95% CI 2.4-3.5; agency B: 34%, *P*<.001, OR 1.5, 95% CI 1.2-1.8 vs face-to-face: 27%), blood in feces (agency A: 26%, *P*<.001, OR 4.2, 95% CI 3.3-5.6; agency B: 17%, *P*<.001, OR 2.1, 95% CI 1.6-2.7 vs face-to-face: 9.6%) and tiredness (agency A: 28%, *P*<.001, OR 2.1, 95% CI 1.7-2.7; agency B: 22%, *P*=.04, OR 1.3, 95% CI 1.0-1.6 vs face-to-face: 16%). Online samples were more likely to answer “don’t know” when asked to recall warning signs for cancer than face-to-face responders (agency A: 1.8%, *P*=.02, OR 4.4, 95% CI 1.5-19.3; agency B: 6.1%, *P*<.001, OR 20.4, 95% CI 7.5-38.5; face-to-face: 0.2%).

#### Recognition of Cancer Signs

Agency A participants demonstrated greater recognition of signs and symptoms, recognizing a mean of eight of nine presented signs and symptoms of cancer, compared with ONS and agency B participants who recognized a mean of seven.

An unexplained lump or swelling was the most commonly recognized sign in all samples (face-to-face: 94.7%; agency A: 98.4%; agency B: 94.7%; Table 2). Agency A participants were more likely than face-to-face participants to recognize a lump (98% vs 95%, *P*=.009, OR 2.4, 95% CI 1.3-4.6) and unexplained weight loss (96% vs 89%, *P*<.001, OR 3.5, 95% CI 2.3-5.5). For other signs, there were no significant differences between agency A and face-to-face responses.

Agency B participants were less likely than face-to-face participants to recognize a lump (94% vs 95%, *P*=.002, OR 0.4, 95% CI 0.3-0.7), changes in bowel habits (88% vs 90%, *P*<.001, OR 0.5, 95% CI 0.4-0.7), persistent cough (83% vs 84%, *P*=.01, OR 0.7, 95% CI 0.6-0.9), unexplained weight loss (87% vs 89%, *P*=.03, OR 0.7, 95% CI 0.5-0.9), persistent difficulty swallowing (76% vs 78%, *P*=.004, OR 0.7, 95% CI 0.6-0.9), and unexplained bleeding (86% vs 88%, *P*=.005, OR 0.7, 95% CI 0.4-0.9). Agency B participants were more likely to recognize a sore that does not heal as a sign or symptom of cancer (70% vs 63%, *P*=.01, OR 1.3, 95% CI 1.1-1.5).

**Table 2:** Percentage of participants from each sample who answered “yes” to the question “Do you think that the following could be a warning sign for cancer?” (N=4075).

Yes, it could	Face-to-face, %	Online			
	Office for National Statistics (n=819)	Agency A (n=1190)		Agency B (n=2066)	
		Participants, %	*P* value	Participants, %	*P* value
Unexplained lump or swelling	94.7	98.4	.009	94.7	.002
Change in appearance of a mole	92.9	95.9	.09	93.9	.006
Persistent change in bowel or bladder habits	89.8	91.4	.58	88.2	.001
Unexplained weight loss	89.1	96.4	<.001	86.5	.03
Unexplained bleeding	88.0	89.1	.93	86.3	.005
Persistent cough or hoarseness	83.7	86.7	.10	82.8	.01
Persistent unexplained pain	79.0	82.0	.05	83.8	.47
Persistent difficulty swallowing	78.3	76.3	.15	76.2	.004
Sore that does not heal	63.0	66.6	.18	70.0	.01

### Awareness of Risk Factors for Cancer

#### Recall of Cancer Risk Factors

Agency A participants recalled a mean of five risk factors compared with both face-to-face and agency B participants who recalled a mean of three. Fewer agency A participants recalled zero risk factors (3.2%) than face-to-face (8.2%) or agency B (11.6%) participants (Multimedia Appendix 1).

The most frequently recalled risk factor within all samples was smoking, but recall was significantly lower in the agency B sample (*P*<.001, OR 0.4, 95% CI 0.3-0.5; Table 3). The same pattern was seen for alcohol (agency A: 55%, *P=*.07, OR 1.2, 95% CI 1.0-1.4; face-to-face: 54%; agency B: 43%, *P*<.001, OR 0.7, 95% CI 0.6-0.8). A higher proportion of agency B participants answered “don’t know” to this recall question (5.4%, *P*<.001; face-to-face: 0.1%; agency A: 0.9%).

**Table 3:** Recall of risk factors for cancer from the three samples (N=4075).

Risk factor	Face-to-face, %	Online			
	Office for National Statistics (n=819)	Agency A (n=1190)		Agency B (n=2066)	
		Participants, %	*P* value	Participants, %	*P* value
Smoking	81.9	81.5	.96	68.6	.001
Alcohol	53.5	55.1	.07	43.3	.001
Diet (unspecified)	36.2	50.3	.001	40.0	.02
Sunburn	25.0	30.1	.001	21.1	.06
Being overweight	14.9	20.0	.04	25.6	.001
Exercise	13.8	24.1	.001	16.4	.06
Occupational exposure	13.7	12.2	.88	8.5	.001
Genes	11.5	23.8	.001	19.8	.001
Pollution	10.4	13.0	.002	7.8	.02
Family history	10.0	22.6	.001	15.2	.003
Lifestyle	9.6	18.1	.001	14.9	.02
Stress	8.4	11.7	.001	5.6	.02
Radiation	5.9	6.3	.19	4.7	.06
High-fat diet	4.6	2.1	.001	1.0	.001
Red meat	3.7	3.9	.51	3.3	.80
Sun beds	3.7	5.3	.09	2.0	.01
Passive smoking	2.7	3.1	.07	1.5	.001
Older age	2.3	6.1	.001	4.8	.009
Mobile phones	1.1	0.3	.60	0.2	.06
Many sexual partners	1.0	0.6	.98	1.5	.10
Other	12.9	24.5	.001	12.9	.61
Nothing	2.8	0.0	—	0.4	—
Refused	4.4	0.0	—	0.0	—
Don’t know	0.1	0.9	.05	5.4	.001

Recall of sunburn (30%, *P*<.001, OR 1.7, 95% CI 1.3-2.0), genes (24%, *P*<.001, OR 2.4, 95% CI 1.8-3.0), and lack of exercise (24%, *P*<.001, OR 2.1, 95% CI 1.6-2.7) as risk factors was significantly higher in the agency A survey compared with the face-to-face survey (sunburn: 25%; genes: 12%; lack of exercise: 14%). Agency B participants were less likely than face-to-face participants to recall occupational exposure (9% vs 14%, *P*<.001, OR 0.6, 95% CI 0.5-0.8), stress (6% vs 8%, *P*=.02, OR 0.6, 95% CI 0.5-0.9), and high-fat diet (1% vs 5%, *P*<.001, OR 0.2, 95% CI 0.1-0.3). Participants from both online surveys were more likely than face-to-face participants to recall being overweight (agency A: 20%, *P*=.04, OR 1.4, 95% CI 1.1-1.8; agency B: 25%, *P*<.001, OR 1.8, 95% CI 1.4-2.2; face-to-face: 15%), family history (agency A: 23%, *P*<.001, OR 2.8, 95% CI 2.1-3.6; agency B: 15%, *P*=.003, OR 1.5, 95% CI 1.1-2.0; face-to-face: 10%), lifestyle (agency A: 18%, *P*<.001, OR 1.8, 95% CI 1.4-2.4; agency B: 15%, *P*=.02, OR 1.4, 95% CI 1.1-1.8; face-to-face: 10%), diet (agency A: 50%, *P*<.001 OR 2.2, 95% CI 1.8-2.7; agency B: 40%, *P*=.02, OR 1.2, 95% CI 1.0-1.4; face-to-face: 36%), and older age (agency A: 6%, *P*<.001, OR 3.4, 95% CI 2.1-5.9; agency B: 5%, *P*=.009, OR 2.0, 95% CI 1.2-3.5; face-to-face: 2%) as risk factors for cancer. The only risk factor that face-to-face participants were more likely to recall was having a high-fat diet (face-to-face: 5%; agency A: 2%, *P*=.001, OR 0.4, 95% CI 0.3-0.7; agency B: 1%, *P*<.001, OR 0.2, 95% CI 0.1-0.3).

### Recognition of Cancer Risk Factors

Online participants recognized more risk factors, a mean of 9 of 12 listed compared with 8 for face-to-face participants.

Participants recruited by online agencies were more likely than face-to-face participants to recognize being overweight (agency B: 74%, *P*<.001, OR 1.4, 95% CI 1.1-1.7; agency A: 73%, *P*=.004, OR 1.3, 95% CI 1.1-1.7; face-to-face: 67%), having a family history of cancer (agency B: 77%, *P*=.02, OR 1.3, 95% CI 1.0-1.5; agency A: 77%, *P*<.001, OR 1.5, 95% CI 1.2-1.9; face-to-face: 69%), eating too much red or processed meat (agency B: 61%, *P*<.001, OR 1.5, 95% CI 1.3-1.8; agency A: 58%, *P*<.001, OR 1.5, 95% CI 1.3-1.8; face-to-face: 52%), and infection with human papillomavirus (HPV) as risk factors of cancer (agency A: 41%, *P*<.001, OR 1.8, 95% CI 1.5-2.2 agency B: 49%, *P*<.001, OR 2.4, 95% CI 2.0-2.9; face-to-face: 29%). Agency B participants were more likely than face-to-face participants to recognize older age (68% vs 60%, *P*<.001, OR 1.4, 95% CI 1.2-1.7) but less likely to recognize smoking (95% vs 96%, *P*=.001, OR 0.4, 95% CI 0.2-0.7) as risk factors of cancer (Table 4).

**Table 4:** Percentage of participants from each sample that recognized each risk factor for cancer (N=4075).

Risk factor	Face-to-face, %	Online			
	ONS (n=819)	Agency A (n=1190)		Agency B (n=2066)	
		Participants, %	*P* value	Participants, %	*P* value
Smoking	96.3	98.6	.24	95.4	.001
Getting sunburned	94.0	94.6	.40	93.7	.42
Exposure to another person’s smoking	88.6	88.2	.77	86.1	.002
Drinking alcohol	78.9	78.6	.43	78.6	.93
Having a close relative with cancer	68.5	76.6	.001	76.6	.02
Being overweight	66.6	72.8	.004	74.1	.001
Being older	60.1	57.1	.16	67.8	.001
Not eating many fruits and vegetables	52.8	53.3	.95	53.6	.13
Not eating enough fiber	52.6	46.4	.10	49.1	.37
Eating too much red or processed meat	51.5	57.9	.001	61.0	.001
Not doing much physical activity	49.7	56.1	.002	55.1	.001
Infection with HPV (human papillomavirus)	29.2	41.3	.001	48.9	.001

### Barriers to Seeing a General Practitioner

**Figure 2:**
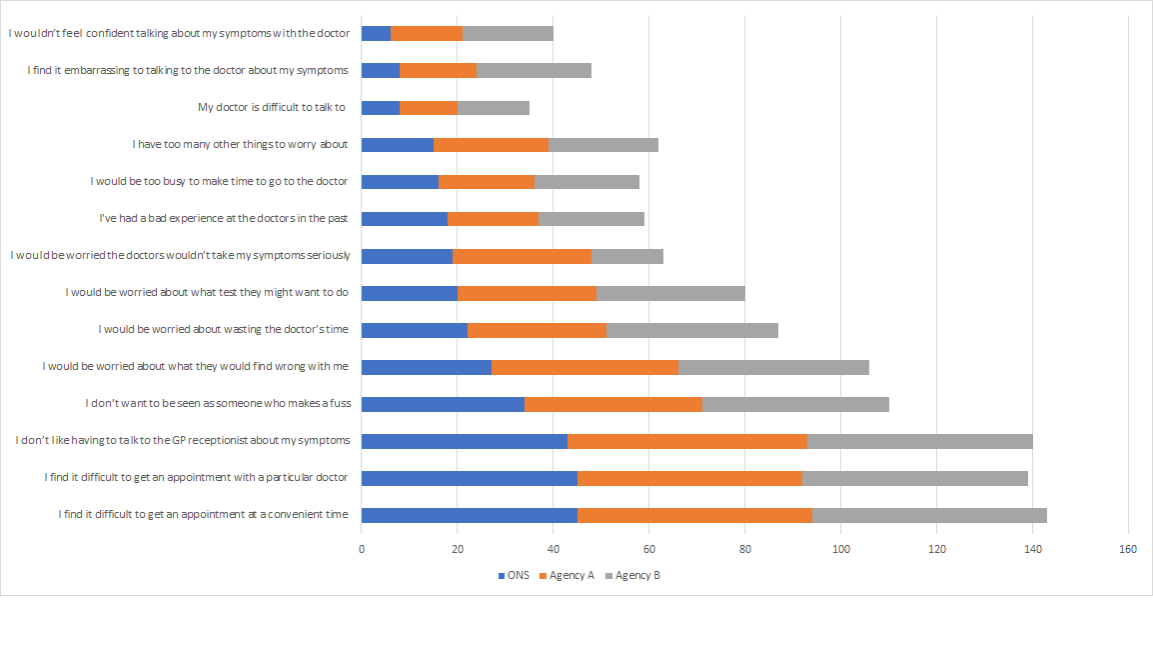
Percentage of participants that endorsed barriers to going to the doctor.

Online survey participants were significantly more likely to endorse 8 of 14 barriers to seeing a GP than face-to-face participants. The most frequently endorsed barrier for face-to-face and agency B participants was “I find it difficult to get an appointment at a convenient time”; for agency A participants, it was “I don’t like having to talk to the GP receptionist.” Agency B participants were more likely than face-to-face participants to endorse an additional barrier “my doctor is difficult to talk to” (*P*=.001, OR 1.6, 95% CI 1.2-2.1). Figure 2 shows the percentage of participants that endorsed barriers to going to the doctor.

### Interactions Between Outcomes and Demographic Variables.

Recall of bleeding or blood loss, cough, and difficulty swallowing showed significant interactions between sex and survey provider. For participants living in Scotland, those recruited by agency B were significantly less likely to recall bleeding or blood loss as a sign of cancer compared with those recruited by agency A (*P*=.04).

Fewer females recognized family history as a risk factor of cancer when completing face-to-face interviews than in online surveys (agency A females: *P*=.006; agency B females: *P*<.001). Significantly fewer males recognized not doing enough physical exercise as a risk factor of cancer in the agency B survey compared with agency A (*P*=.02).

## Discussion

### Analysis

This analysis explored the viability of moving from face-to-face to online data collection for the Cancer Research UK’s CAM.

### Principal Results

Comparisons of data collected using face-to-face interviews and online surveys revealed minor differences between samples. Both methods provided broadly representative samples of the UK population with slight differences in awareness of signs, symptoms, and risk factors of cancer and frequency of help-seeking barriers reported, leading us to conclude that online data collection for the CAM is possible.

Recall of certain cancer signs and risk factors varied by demographic group. Recall of bleeding/blood loss, cough, and difficulty swallowing had significant interactions between sex and survey provider. Overall, recognition of risk factors was higher in the online surveys.

Recognition of risk factors varied by sex, education level, and country. Significantly fewer females recognized family history as a risk factor of cancer in the face-to-face survey compared with the online surveys. Significantly fewer males recognized not doing enough physical exercise as a risk factor of cancer in the online samples compared with the face-to-face sample. The reasons for these variations are unclear but provide avenues for further research and action.

Overall, online participants recruited by agency A were significantly more likely to recall cancer signs and risk factors compared with both agency B and face-to-face participants. This finding implies that agency A participants may be more engaged and knowledgeable than the other survey participants. Educational levels did not differ greatly among the three samples. Agency A participants may have been more engaged than other participants because they had previously taken part in a face-to-face survey, indicating that they may be a particularly motivated group.

### Comparison With Prior Work

Previous research has found that levels of awareness of the HPV virus [26] and cholesterol [23] were higher among online than face-to-face or paper survey respondents. In this study, online participants recognized more risk factors than face-to-face participants, including being overweight, having a family history of cancer, eating too much red or processed meat, and infection with HPV (cholesterol was not assessed). However, only one of the online samples reported higher mean recall of risk factors compared with face-to-face participants. This particular panel, agency A, recruited participants after they had taken part in a paper survey, which may have resulted in a more engaged and knowledgeable sample.

Survey research within student populations has suggested that online participants are more likely to answer “don’t know” than those completing the same survey face-to-face [7]. Other research suggests that nonresponse to open-ended questions can be reduced through online data collection [8]. In this study, face-to-face participants were less likely than online participants to respond to recall questions around signs, symptoms, and risk factors with “don’t know.”

Socially desirable behaviors have been found to be less likely to be disclosed in interviews than online questionnaires [27,28]. In this study, online participants were more likely than face-to-face participants to endorse barriers to seeking help. Participants may have found it easier to endorse barriers to visiting the doctor with the context of anonymity afforded by online data collection compared with face-to-face data collection.

### Strengths and Limitations

Although this study provides insights into the possibility of using online data collection for a large representative sample of the UK, there are limitations that warrant consideration. Regarding recruitment, large differences exist in the size of samples recruited online and face-to-face, highlighting the comparative ease of online recruitment. Previous research indicates that online research may not be as representative as face-to-face interviewing [29], but this is often based on the type of recruitment procedures that precede data collection. In this study, both online samples were recruited through panels; however, there may be differences in the ways that panels are recruited and incentivized, which may have affected the results. To mitigate this, each agency employed procedures to ensure their samples were as representative as possible of the Great Britain population.

For the analysis, it was not possible to calculate unique weighting variables, and we relied on those provided by agencies. The questions within each survey were identical; however, there may have been small differences in the presentation of questions within each sample.

It was necessary to limit the demographic variables studied to control the length of the survey, meaning that unobserved differences may have contributed to the differences observed.

It was not possible to compare the samples collected by each survey agency with the Great Britain population data. The Great Britain population data used were publicly available, although confidence intervals were not provided, and statistically significant comparisons were not possible.

It was not possible to access information about response rates or completion times within each sample. This information may have been useful to explore the differences among samples in more depth.

### Conclusions

The relationships between sampling, sample representativeness, survey modality, and subsequent responses are complex. Although sample representativeness varied a little between samples and there are likely unobserved differences, we were encouraged to see that these variations were small overall. This information will be useful in helping us to tailor our recruitment strategy to ensure that we recruit a sample that is as representative as possible of the Great Britain population in future CAM research.

We observed larger differences when looking at responses to the awareness questions themselves, even between the two online samples, which point to the fact that there may be differences in the sampling and running of these panels contributing to these differences.

Nevertheless, the flexibility and potential cost savings of online data collection will enable larger samples and greater variation in content at a lower cost, which will enable the CAM to explore a new and wider range of issues related to the early diagnosis, prevention, and treatment of cancer.

## Appendix

Multimedia Appendix 1 Survey Incentives and Survey Weighting Methodologies.

Multimedia Appendix 2 Number of cancer signs and risk factors recognised and recalled within each sample.

## Abbreviations

CAM: Cancer Awareness Measure
HPV: human papillomavirus
NHS: National Health Service
ONS: Office for National Statistics
