# Supplementary material for: Awareness of the Signs, Symptoms, and Risk Factors of Cancer and the Barriers to Seeking Help in the UK: Comparison of Survey Data Collected Online and Face-to-Face
Source: JMIR Cancer. 2020 Jan 17;6(1):e14539. doi: 10.2196/14539 (PMC6996748; doi:10.2196/14539)
Supplement: Multimedia Appendix 1 [file cancer_v6i1e14539_app1.docx]

**Appendix B**

**Survey Incentives and weighting methodology**

Agency A participants were incentivized in the form of a £5 voucher that could be exchanged at a number of high street stores upon competition of the survey.

Agency B participants are incentivised through a points system. The more surveys they answer, the more points they get. These are then translated into cash once users hit a certain number of points.

**Weighting methodologies**

**Agency A**

Non-response for Agency A’s probability panel surveys can occur at three stages: non-response at the survey used for recruitment, refusal to join the panel at the end of that interview and non-response in the survey of panel members itself. We compute a weight to account for non-response at each of these three stages. The final weight is the product of these three weights. We use this three-stage system because the variables underlying non-response could be different at each stage. With this system we also can maximise the use of all the information available from the face to face survey. These are the three weights we have computed:

1. **Face to face** **survey weight**: the panel members were recruited from a face to face 2015 and 2016 survey. Firstly, the face to face survey weights account for unequal chances of selection in the face to face sampling. Secondly, a non-response model is used to produce a non-response weight. This weight adjusts for non-response at the face to face survey using: region, type of dwelling, whether there were entry barriers to the selected address, the relative condition of the immediate local area, the relative condition of the address, the percentage of owner occupied properties in quintiles and population density. Finally, the face to face 2015 and face to face 2016 weights make the sample of face to face respondents representative of the general British population in terms of gender, age and Government Office Region (GOR).
2. **Panel weight**: this weight accounts for non-response at the panel recruitment stage where some people interviewed as part of the face to face survey chose not to join the panel. A logistic regression model has been used to derive the probability of response of each panel member; the panel weight is computed as the inverse of the probabilities of response. This weight adjusts the panel for non-response using the following variables: age and sex groups, GOR, face to face year, household type, household income, education level, internet access, ethnicity, tenure, social class group, economic activity, political party identification, and interest in politics. The resulting panel weight has been multiplied by the face to face 2015 and 2016 weights, so the panel is representative of the population.
3. **Survey weight**: this weight is to adjust the bias caused by non-response to this particular panel survey. A logistic regression model has been used to compute the probabilities of response of each participant. The panel survey weight is equal to the inverse of the probabilities of response. The initial set of predictors used to build the model was the same as for the panel weight; and at this wave the final set of variables used was also the same. The final survey weight is the result of multiplying the survey weight by the compounded panel weight.

**Agency B**

All reputable research agencies weight data as a fine-tuning measure and at agency B we weight by age, gender, social class, region, level of education, how respondents voted at the previous election, how respondents voted at the EU referendum and their level of political interest. Targets for the weighted data are derived from four sources:

1. The census
2. Large scale random probability surveys, such as the Labour Force Survey, The National Readership survey and the British Election Study
3. The results of the 2015 general election.
4. Official ONS population estimates

**ONS**

Despite the considerable efforts made by interviewers to maximize response rates, a proportion of selected individuals decline to take part or cannot be contacted. In order to compensate for possible non-response bias, the Omnibus sample is divided into weighting classes of age-group by sex and Government Office Region. Population data for these sub-groups are provided by ONS, and survey data are grossed to population totals within these sub-groups
