# Supplementary material for: Awareness of the Signs, Symptoms, and Risk Factors of Cancer and the Barriers to Seeking Help in the UK: Comparison of Survey Data Collected Online and Face-to-Face
Source: JMIR Cancer. 2020 Jan 17;6(1):e14539. doi: 10.2196/14539 (PMC6996748; doi:10.2196/14539)
Supplement: Multimedia Appendix 2 [file cancer_v6i1e14539_app2.docx]

| Number of signs and symptoms recalled across each sample | | | | | | | | | | | | | |
| --- | --- | --- | --- | --- | --- | --- | --- | --- | --- | --- | --- | --- | --- |
|  | **0** | **1** | **2** | **3** | **4** | **5** | **6** | **7** | **8** | **9** | **10** | **11** | **12** |
| **ONS** | 9.3% (76) | 12.3% (101) | 17.8% (146) | 24.2% (198) | 17.6% (144) | 9.0% (74) | 5.3% (43) | 3.2% (26) | 0.7% (6) | 0.0% (0) | 0.2% (2) | 0.1% (1) | 0.2%  (2) |
| **Agency A** | 3.8% (45) | 4.2% (50) | 6.5% (77) | 12.0% (143) | 15.1% (180) | 14.3% (170) | 16.1% (191) | 10.3% (122) | 6.5% (77) | 4.1% (49) | 3.7% (44) | 1.5% (18) | 1.3%  (15) |
| **Agency B** | 14.0% (289) | 10.0% (206) | 13.8% (285) | 18.1% (374) | 16.6% (343) | 11.2% (232) | 8.6% (178) | 2.6% (53) | 2.3% (48) | 1.3% (27) | 0.8% (16) | 0.6% (13) | 0.1%  (2) |
| Number of signs and symptoms recognised across each sample | | | | | | | | | | | | | |
|  | 0 | 1 | 2 | 3 | 4 | 5 | 6 | 7 | 8 | 9 | - | - | - |
| ***ONS*** | 2.1% (17) | 0.7% (6) | 1% (8) | 1% (8) | 1.7% (14) | 4.9% (40) | 6.3% (52) | 12% (98) | 24.9% (204) | 45.4% (372) |  |  |  |
| **Agency A** | 0.3% (4) | 0.1% (1) | 0.3% (3) | 0.5% (6) | 1.8% (21) | 2.7% (32) | 7.7% (92) | 12.5% (149) | 19.7% (235) | 54.4% (647) |  |  |  |
| **Agency B** | 3.4% (70) | 0.3% (7) | 1.1% (22) | 1.6% (34) | 2.5% (51) | 3.8% (79) | 6.6% (136) | 10.5% (217) | 13.3% (274) | 56.9% (1176) |  |  |  |
| Number of risk factors of cancer recalled across each sample | | | | | | | | | | | | | |
|  | **0** | **1** | **2** | **3** | **4** | **5** | **6** | **7** | **8** | **9** | **10** | **11** | **12** |
| **ONS** | 8.2% (67) | 9.9% (81) | 15.4% (126) | 21.7% (178) | 23.2% (190) | 10.6% (87) | 7.0% (57) | 2.4% (20) | 1.0% (8) | 0.6% (5) | 0.0% (0) | 0.0% (0) | 0.0% (0) |
| **Agency A** | 3.2% (38) | 2.9% (34) | 6.8% (81) | 11.4% (136) | 16.9% (201) | 18.3% (218) | 17.7% (211) | 9.6% (114) | 5.5% (66) | 3.6% (43) | 2.1% (25) | 1.6% (19) | 0.3% (3) |
| **Agency B** | 11.6% (239) | 7.2% (148) | 11.6% (240) | 18.7% (386) | 19.1% (394) | 13.4% (277) | 11.2% (231) | 2.4% (49) | 2.3% (48) | 1.7% (35) | 0.4% (9) | 0.2% (4) | 0.3% (6) |
| Average number of risk factors of cancer recognised across each sample | | | | | | | | | | | | | |
|  | **0** | **1** | **2** | **3** | **4** | **5** | **6** | **7** | **8** | **9** | **10** | **11** | **12** |
| **ONS** | 2.2% (18) | 0.5% (4) | 0.6% (5) | 4.3% (35) | 6% (49) | 7.6% (62) | 8.7% (71) | 14.5% (119) | 11% (90) | 12.2% (100) | 13.8% (113) | 11.5% (94) | 7.2% (59) |
| **Agency A** | 0.2% (2) | 0.4% (5) | 1.2% (14) | 1.4% (17) | 4.5% (53) | 6.9% (82) | 8.7% (103) | 12.4% (148) | 9.5% (113) | 12.4% (147) | 14.1% (168) | 14.5% (173) | 13.9% (165) |
| **Agency B** | 2.9% (59) | 0.6% (13) | 1.3% (26) | 2.4% (49) | 4.9% (102) | 6.7% (139) | 7.5% (155) | 8.8% (181) | 9.4% (194) | 9.8% (203) | 10.5% (216) | 12.2% (252) | 23.1% (477) |
